# Usability of a virtual reality circle drawing task to assess upper-limb motor performance in children and young people with cerebral palsy: pilot study

**DOI:** 10.1186/s12887-026-06626-8

**Published:** 2026-02-24

**Authors:** Mohammed M. Alrashidi, Jack O. Evans, Richard J. Tomlinson, Craig A. Williams, Gavin Buckingham

**Affiliations:** 1 Physical Therapy Department, College of Medical Rehabilitation Sciences, Taibah University, Madinah, Saudi Arabia; 2 Public Health and Sport Sciences, University of Exeter, Exeter, UK; 3 Children’s Health and Exercise Research Centre, University of Exeter, Exeter, UK; 4 Royal Devon University Healthcare NHS Foundation Trust, Exeter, UK

**Keywords:** Technology-based assessment, Paediatric rehabilitation, Virtual environment, Upper-limb motor skills

## Abstract

**Background:**

Cerebral palsy (CP) is a major cause of upper-limb impairments. Immersive virtual reality (iVR) is emerging as an adjunct to traditional CP rehabilitation methods. However, the potential utility of iVR for assessing upper -limb motor function in CP remains unexplored. The aim of this study was to evaluate the usability of an iVR circle drawing task in assessing the upper-limb motor function of children with CP.

**Methods:**

Nine children with CP (age: 13 ± 2.9 y) completed an iVR circle drawing task delivered through a Meta-Quest-2 headset. Outcomes were children’s rating of the iVR task in the System Usability Scale (SUS), in addition to circle drawing metrics (movement time, mean velocity and circle roundness) derived from the position of the controllers during the task, which were correlated with scores from the Box and Block Test (BBT) and the Duruoz Hand Index (DHI).

**Results:**

The average score for the SUS was 74, indicating good usability and acceptability. No adverse effects were reported by participants. Strong positive correlations were found between the BBT scores and mean velocity (rho = 0.78, *p* = 0.01) and roundness (rho = 0.92, *p* < 0.001) scores in the circle drawing task. Strong negative correlations were also observed between the DHI scores and mean velocity (rho=-0.82, *p* = 0.007) and roundness (rho=-0.75, *p* = 0.02) scores.

**Conclusions:**

This study shows that the iVR circle drawing task is a usable tool to capture upper-limb motor performance of children with CP, highlighting the value of clinical development of iVR in CP rehabilitation.

**Supplementary Information:**

The online version contains supplementary material available at 10.1186/s12887-026-06626-8.

## Background

Cerebral palsy (CP) describes a group of heterogeneous impairments affecting children, which impact their daily activities, independence and well-being [1]. Upper-limb impairments are common amongst those who have CP, with a reported prevalence of 83% among affected children [2]. In physiotherapy practice, the successful assessment of upper-limb function is key for setting treatment plans, evaluating progress, and achieving satisfactory therapeutic outcomes [3]. Examples of valid and reliable upper-limb assessment tools include the Quality of Upper Extremity Skills Test [4], the Jebsen-Taylor Hand Function Test [5], the Nine Hole Peg Test [6], and universal goniometer [7]. However, these tools have a number of shortcomings such as the lack of granularity in scoring systems [8] and potential ceiling effects [9, 10]. Consequently, recent guidelines in CP management have shown that using technology-based assessments offers innovative solutions to overcome challenges related to conventional assessment tools [11].

Immersive virtual reality (iVR) is attracting considerable attention in physiotherapy and rehabilitation [12]. This technology enables users to experience and interact with virtual environments in three-dimensional space. Typically, iVR is provided through commercially available head-mounted displays (HMDs), such as the Meta Quest series of headsets. The iVR HMDs have built-in motion capture functions that accurately quantify hand and head movements [13]. These features of iVR HMDs could open new avenues for the potential of this technology in physiotherapy beyond promoting therapeutic exercises, aiming for other therapeutic goals, such as assessments. Indeed, the use of iVR as an upper-limb assessment tool for stroke survivors has shown promising outcomes [14, 15].

However, there are no data on the usability of iVR as an upper-limb assessment tool for children and young people with CP. The few studies that have been conducted using iVR with children have focussed on outcomes often unrelated to upper-limb function. For example, Ammann-Reiffer, Kläy [16] examined the usability of iVR to promote walking activities in children with neurological conditions, and Hocking, Ardalan [17] assessed the usability of iVR for motor proficiency in youths with autism spectrum disorders. Some studies have investigated the usability of iVR HMDs as a home-based upper-limb intervention for paediatric patients [18], as well as the use of virtual environments and wearable haptic devices for children with CP and dyspraxia [19]. These studies provided preliminary evidence that iVR may be a useful tool for children, however they did not evaluate its usability to aid upper-limb motor assessment. Therefore, there is a need to examine the usability of this technology as an upper-limb motor assessment tool for children with CP.

Circle drawing is a simple task that requires interaction between sensorimotor, perceptual, and cognitive systems [20, 21]. Previous research has investigated circle drawing task in children using digitizing tablets [20, 22, 23]. These studies showed the potential value of this task in quantifying youth motor performance. Recently, Evans and Colleagues [24] developed an iVR circle drawing task, which was administered through a Meta-Quest 2 HMD and allowed the researchers to distinguish the dominant and non-dominant hands of healthy adults. This work was followed up by a study by Alrashidi, Evans [25], who employed this iVR circle drawing task in typically-developing children to assess the usability of this task to capture upper-limb motor performance in this population. Collectively, these studies showed promising results in terms of the usability and acceptability of iVR circle drawing to assess upper-limb motor function in a range of contexts.

The goal of this study was to evaluate the viability of the iVR circle drawing task developed by Evans, Tsaneva-Atanasova [24] for capturing the upper-limb motor performance of children with CP. Therefore, the aims of this study were:

- To assess the usability of the circle drawing task delivered by an iVR headset to quantify motor performance.
- To identify any adverse effects of using headsets with participants.
- To test the relationships between usability scores, upper limb motor performance (captured by the Box and Block test and Duruöz Hand Index scores) and circle drawing metrics in iVR.

## Methods

This study was approved by the National Health Services (NHS) Health Research Authority (IRAS ref. 330082) and the Research Ethics Committee of the University of Exeter (ref. 1532843). Additionally, permission was obtained from the local NHS Hospital Trust Research and Development (RDUH - R&D – ref. 2407584).

### Participants

**Fig. 1:**
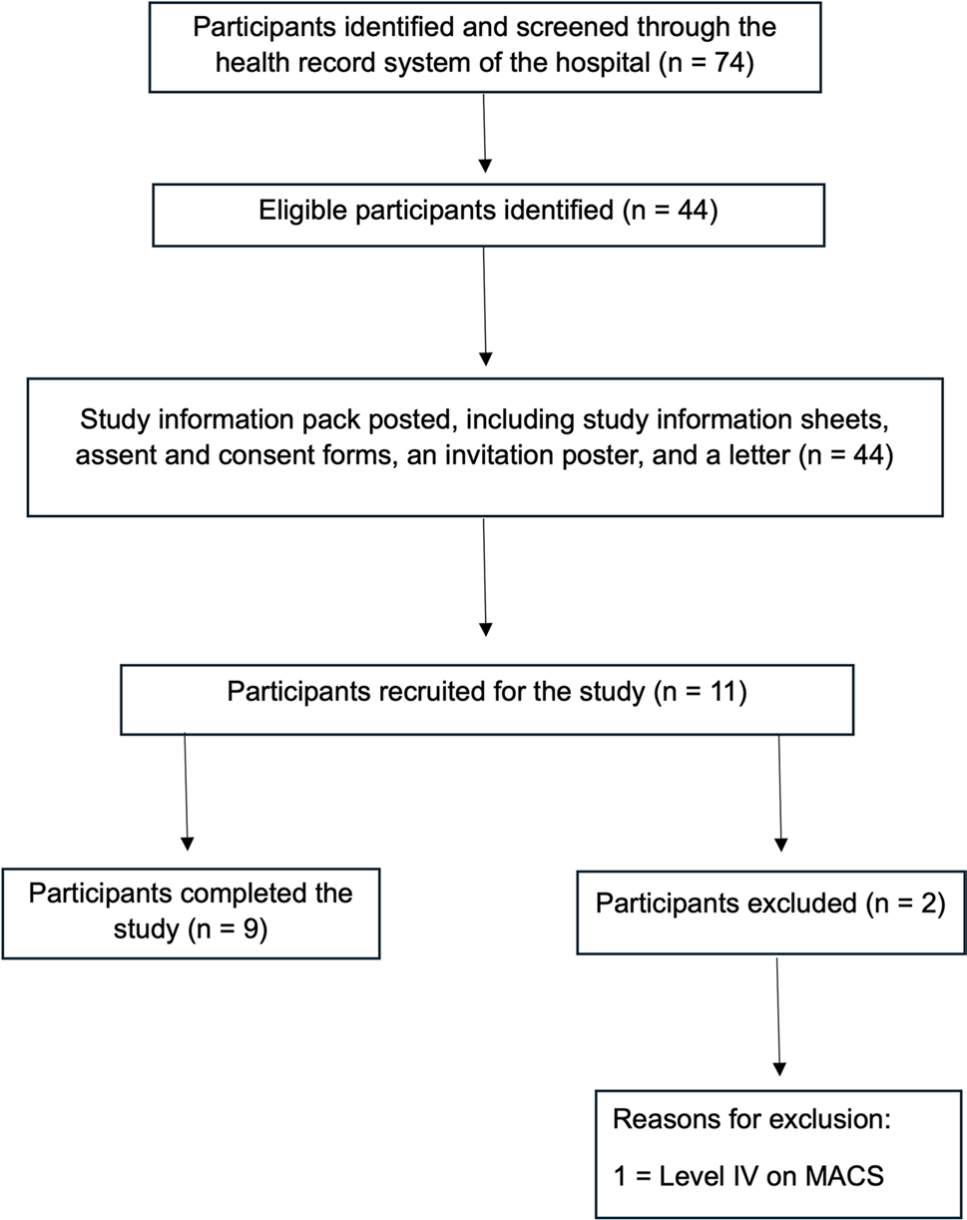
Flowchart of participant identification and recruitment

Eleven children and young people with CP were recruited, of whom nine completed the study. The participant recruitment flowchart is presented in Fig. 1. Potential participants were identified from the hospital registry at the Royal Devon University Healthcare NHS Trust. Following screening of medical records, individuals who did not meet the predefined inclusion criteria were excluded. Eligible families were subsequently approached through invitations sent home via the clinical service. Non-response to these invitations accounted for the majority of participant loss between screening and recruitment. Participants who responded and met all eligibility criteria were invited to attend laboratory-based testing sessions.

The inclusion criteria were as follows: (1) children and young people (both female and male) with CP (2) aged between 10 and 17 years (from their 10th birthday to the day before their 18th birthday) (3) manual ability equivalent to levels I-III on Manual Ability Classification System (MACS) and (4) no cognitive or visual disorders that might impede participation (Parent/carer-reported). Children with CP were excluded if they have head injuries, severe cognitive and/or vision difficulties, or medical history of epilepsy or nonfebrile seizures.

### Screening tools

‘Functional classifications’ for CP is a central concept in clinical practice that involves categorising CP based on functional independence, particularly in terms of gross motor function and fine motor function [26]. The Gross Motor Function Classification System (GMFCS) and the MACS are common standardised screening tools that aim to assess the functional profile of children with CP by looking at what activities they can do and how they participate and engage with daily activities, as described in the World Health Organization’s [27] International Classification of Functioning, Disability, and Health (Children & Youth version - ICF-CY). Despite the expected variations in gross and manual abilities among children with CP, both tools should be reported to provide a holistic profile for individuals with CP [28]. Therefore, all participants in this study were screened using GMFCS and MACS.

### Experimental procedures and outcome measures

The experimental procedures for this study were conducted during a single visit to a research laboratory at the University of Exeter. All participants attended the laboratory accompanied by a parent or carer. For participants younger than 16 years of age, written informed consent was obtained from parents or legal guardians, and written assent was obtained from the participants. Participants aged 16 years and older provided their own written informed consent prior to participation. Participants were then physically screened to confirm that they met the inclusion criteria. Following screening, verbal instructions outlining all study procedures were provided before participants donned a Meta Quest-2 virtual reality headset (Meta Platforms, Inc., California, USA) to complete the VR circle-drawing task.

The VR task used in this study was a circle drawing task developed in the Unity game engine (Unity Technologies, San Francisco, USA) by Evans, Tsaneva-Atanasova [24], and then refined for children and adolescents [25]. The VR environment in this task involves a 360º virtual room comprised of a monitor screen presenting instructions on how to complete the task and a virtual holographic circle. The virtual circle had a small start point to show where to start and end the drawing movement. This semi-transparent circle had a small start point to show where to start and end the drawing movement, and remained visibit throughout the task to provide a consistent visual guide for participants.The participants were instructed to accurately trace the outline of the circle shown in their display using the hand-held controller. To draw a circle, participants placed the controller over the start point, held the trigger down and trace the circle to the instructed direction (right or left) until reaching the start point again (Supplementary video 1). During this period, a complete drawing movement was recorded as one trial. If the participants released the trigger before reaching the start point, the trial automatically restarted. A non-visible boundary around VR circle was used to prevent participants from making movements that were not task-directed (i.e., drawing upwards away from the circle). This boundary was 7 cm in each direction. If a participant’s movement crossed this boundary, a prompt message requested the participant to redo the trial more accurately.

The iVR circle drawing task used in this study involved three stages: a demonstration video, followed by practice trials, and the experimental trials. Before starting the experiment, the participants were allowed to adjust the distance and the height of the virtual monitor and the circle to their body to ensure a comfortable position while conducting the experiment without physical discomfort. Each participants watched a short demonstration video showing how to complete the tasks correctly and then performed six practice trials (three with each hand) to familiarise themselves with the task. Following this, the participants proceeded to the experimental trials, which consisted of 16 trials (8 circles with each hand in a randomised order). As the task was self-paced, the participants were able to rest between trials as long as was needed. Participants drew the circles with one hand at a time and were instructed to draw circles with the right hand in a clockwise direction, and circles with the left hand in a counterclockwise direction. During the experiment, the participants received visual and audio feedback in the form of a humming sound and a cyan coloured line appeared on the participants’ hand path to indicate drawing. After releasing the trigger, the humming sound stopped, and the coloured line disappeared. The participants conducted the experiment either standing or sitting as they preferred, in a single session lasting up to 30 min, and a researcher supervised them throughout the protocol.

After completing the iVR circle drawing trials, the participants were asked to remove the HMD and then complete a pencil-and-paper version of an adapted System Usability Scale (SUS), which is a short reliable 5 point Likert scale that assesses a device or system usability and learnability [29]. The SUS comprises ten statements concerning the learnability (statements 4 and 10), ease of use (statement 3), confidence (statement 9), and remaining statements appraise the usability. These statements were adapted by adding terms related to iVR HMDs and children. The ten statements are structured in both negative (even-numbered statements) and positive (odd-numbered statements) forms [30], and are rated between zero and four (zero = strongly disagree and four = strongly agree). The total score out of these statements for each patient is multiplied by 2.5 to obtain an overall score of 100. The overall score is interpreted as: not acceptable (zero–64), acceptable (65–84), or highly acceptable (85–100) [30]. As part of the usability assessment, safety considerations focused on adverse effects associated with the iVR use (e.g., dizziness, headache, vomiting, postural strain) while using the headset. At the end of the SUS questionnaire, there were two closed questions about these adverse effects associated with iVR headset use.

After completing the SUS, participants performed the Box and Block Test (BBT). The BBT is simple and quick-to-administer assessment of unilateral manual dexterity of children with CP [31, 32]. It has been shown that the BBT is valid (*r* = 0.78) and reliable (intraclass correlation coefficient = 0.98) tool for assessment of function in children with CP [33, 34]. The instructions, set up and scoring of the BBT in this study were as described by Mathiowetz, Federman [34]. The BBT kit includes a wooden box with a partition in the middle, 150 coloured blocks in one compartment (faced the tested side) and a stopwatch. The BBT kit was placed on a table and patients were seated and had 15-second trial for each hand to familiarise themselves with the test. Patients were instructed to begin the assessment using their dominant hand, picking up one block at a time, transferring it over a partition, and promptly releasing it into the other compartment within a 60-second timeframe. Thereafter, the same procedure was repeated with the non-dominant hand. A researcher closely monitored and timed the assessment with the stopwatch. The evaluation involved scoring patients based on the number of blocks successfully moved from one compartment to the other within the allotted minute. If two blocks moved at once, it was counted as one block and if the block bounced out of the box it was counted. Each hand was assessed separately, then both hands scores were averaged to obtain a total score in which higher scores indicate better manual dexterity [34].

Following this, the participants were asked to complete a Duruoz hand index (DHI) questionnaire with parents’ assistance if needed. The DHI is a subjective patient-reported survey that evaluates the manual ability and function during activities of daily living [35]. It has been reported that DHI is a valid (*r* = 0.84) and reliable (intraclass correlation coefficient = 0.94) hand tool of children with CP [36]. The DHI is comprised of 18 questions concerning the hand use in daily tasks of five domains: (a) in the kitchen (8 questions), (b) dressing (2 questions), (c) hygiene (2 questions), (d) in the school (2 questions) and (e) other (4 questions). The individuals questions are scored on a 6-point Likert scale (0 = without difficulty, 5 = impossible), and then the individual score of the questions is summed to obtain a total score [36]. The total score of the DHI ranges between 0 and 90, and lower score indicate better manual ability in activities of daily living [36].

### Circle drawing kinematic metrics

Previous research has shown that movement time and mean velocity of circle drawing performance are key factors in assessing the fine motor development and skills [20, 22]. In addition, it has been reported that these kinematic metrics are reflective of the individual ability of motor control of delicate tasks, such as drawing [37]. Besides, it has been demonstrated that drawing rounded circles is linked with better performance on conventional outcome measures, e.g., Fugl-Meyer test [38]. Evans, Tsaneva-Atanasova [24] was able to distinguish dominant and non-dominant hand performance in this iVR circle drawing task based on movement time and movement velocity. Therefore, movement time, mean velocity and circle roundness were captured for each iVR trial as circle drawing kinematic metrics [20, 22, 24].

### Data analysis

#### iVR data processing

A custom C# script conducted in Unity recorded the XYZ positions and rotations of the controllers of each hand using the built-in tracking function of the headset. Positional data from the iVR task were captured at a frequency of approximately 72 Hz using this script. Data processing was performed in MATLAB (version R2022a, The MathWorks, Inc., Massachusetts, USA). After filtering data with a dual-pass, zero phase shift Butterworth filter at 10 Hz [39], data were then resampled at 90 Hz to maintain a consistent sampling rate between all trials [24, 40].

An average of movement time and mean velocity of circle drawing were calculated of all trials for each participant using a custom script written in MATLAB. Before averaging and to eliminate resampling artefacts, 1% of each trial’s frames were taken from the movement’s start and end. In addition, in the event that any participant had pressed the trigger but had not yet begun drawing, movement onset was defined as the point where the x-axis velocity exceeded 50 mm/s for three consecutive frames [40, 41].

Circle roundness was defined as the ratio between the major and minor axes of the fitted ellipses [38, 42]. The values of roundness were averaged across the 16 trials for each participant, and range between zero and one, where values closer to one indicate a more perfect circle.

### Statistical analyses

Descriptive statistics (mean, standard deviation, minimum, maximum, frequencies and percents) were used to present demographical data of the patients and scores on BBT, DHI and MACS and GMFCS levels. Adverse effects were narratively reported. Spearman’s correlations were used to assess the strength of relationships between the iVR-derived measures, BBT, DHI, and SUS. A Wilcoxon Signed-Rank Test was conducted to examine if there was a difference in the kinematic measures (i.e., movement time, mean velocity and roundness) between the dominant and non-dominant hands for children with CP. Hand dominance was determined based on self-reported or parent-reported information regarding the hand predominantly used in daily activities. The statistical analyses were conducted in SPSS version 28 (IBM, Chicago, IL, USA).

## Results

The demographic characteristics of the patients are shown in Table 1. No adverse effects associated with the iVR HMDs use were reported for any participants.

**Table 1:** Demographic characteristics of the participants

Sex	5 girls/4 boys
Age (years)^*^	13 ± 2.9
Diagnosis (n)	Hemiplegia (4), quadriplegia (3), diplegia (2)
MACS level (n)	I (2), II (6), III (1)
GMFCS level (n)	I (4), II (2), IV (1), V (2)
Handedness (n)	Right. (7), left. (2)

*Mean and standard deviation; *MACS* Manual Ability Classification System, *GMFCS* Gross Motor Function Classification System, *VR* Virtual Reality

The mean score for the SUS was 74.3 ± 16.2, demonstrating a good level of usability for the task. The descriptive statistics of all outcome measures are summarised in Table 2. A frequency table for the SUS responses is presented in Additional file 1.

**Table 2:** Descriptive statistics of the outcome measures

Outcome measures	Mean ± SD	Min/Max
SUS (/100)	74.3 ± 16.2	50/90
BBT (/50–65)	36.1 ± 11.8	18/52
DHI (/90)	23 ± 20.1	0/51
Movement time (s)^*^	4.3 ± 0.9	3.1/5.5
Mean velocity (mm/s)^*^	25.2 ± 11.1	13.4/40.3
Roundness ratio^*^	0.8 ± 0.2	0.4/0.9

*SD* Standard Deviation, *Min/Max* Minimum/Maximum, *SUS* System Usability Scale, *BBT* Box and Block Test, *DHI* Duruoz Hand Index. *Metrics of iVR-based circle drawing

### Circle drawing metrics and system usability score

**Fig. 2:**
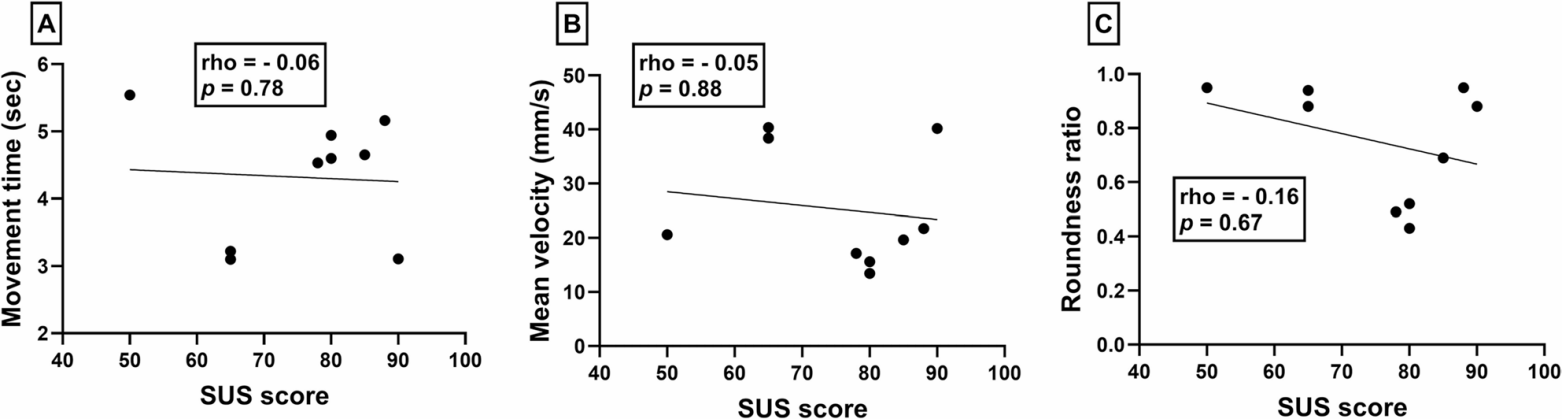
Scatterplots illustrating the relationships between system usability scores (x-axes) and iVR-based circle drawing metrics (y-axes); movement time (**A**), mean velocity (**B**) and roundness ratio (**C**). Data points denote individual participants and depict scores on each outcome measures

Spearman’s correlation revealed no significant correlations between SUS scores and movement time (rho = −0.06, *p* = 0.78 – Fig. 2a), mean velocity (rho = −0.05, *p* = 0.88 – Fig. 2b) or roundness ratio (rho = −0.16, *p* = 0.67 – Fig. 2c).

#### Circle drawing metrics and box and block test

**Fig. 3:**
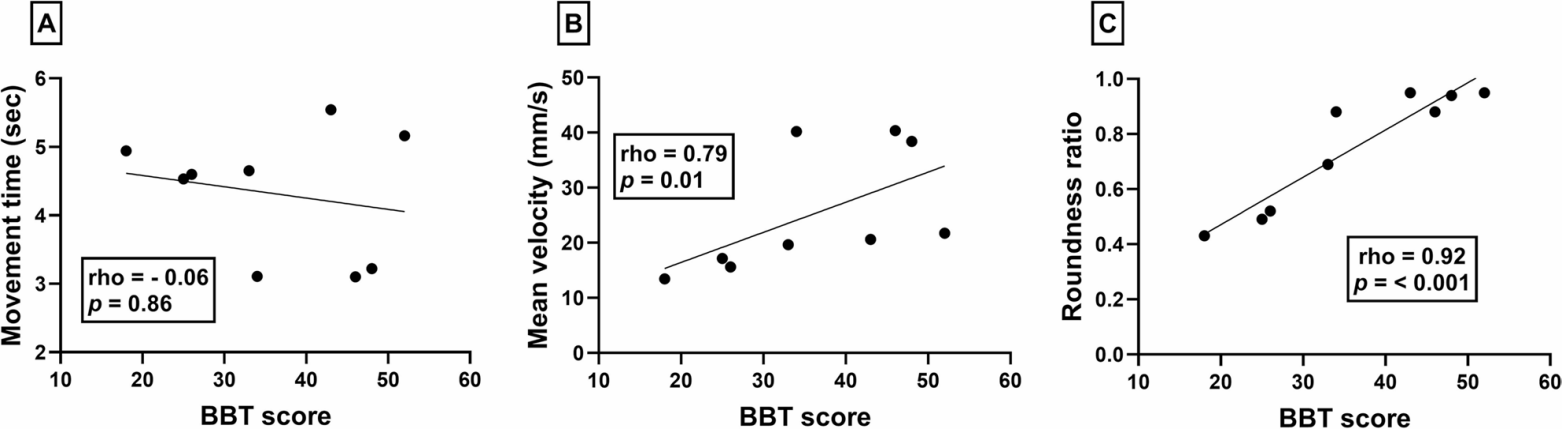
Scatterplots illustrating the relationships between box and block test (x-axes) and iVR-based circle drawing metrics (y-axes); movement time (**A**), mean velocity (**B**) and roundness ratio (**C**). Data points denote individual participants and depict scores on each outcome measures

There was no significant correlation between movement time and BBT scores (rho = −0.06, *p* = 0.86 – Fig. 3a). However, a strong positive correlation was found between mean velocity and BBT scores (rho = 0.78, *p* = 0.01 – Fig. 3b). In addition, a strong positive correlation was observed between roundness ratio and the BBT (rho = 0.92, *p* < 0.001 – Fig. 3c).

#### Circle drawing metrics and duruoz hand index

**Fig. 4:**
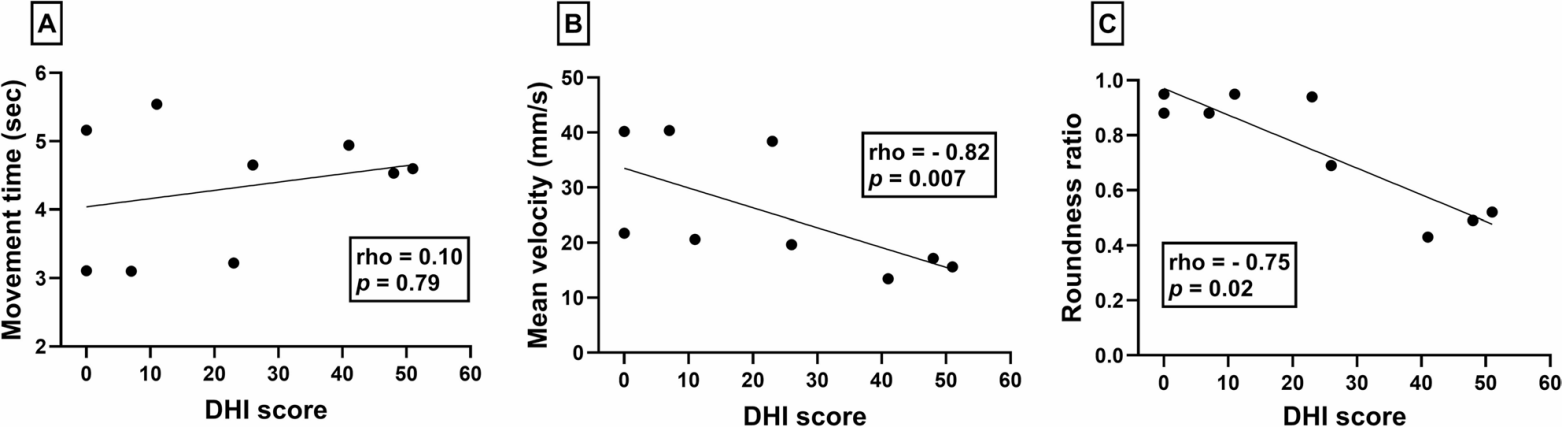
Scatterplots illustrating the relationships between Duruoz hand index (x-axes) and iVR-based circle drawing metrics (y-axes); movement time (**A**), mean velocity (**B**) and roundness ratio (**C**). Data points denote individual participants and depict scores on each outcome measures

No significant correlation was observed between the DHI scores and movement time (rho = 0.10, *p* = 0.79 – Fig. 4a). There were, however, strong negative correlations between the DHI scores and mean velocity (rho = −0.82, *p* = 0.007 - Fig. 4b) and roundness ratio (rho = −0.75, *p* = 0.02 – Fig. 4c).

#### Dominant vs. non-dominant hands

A Wilcoxon Signed-Rank Test revealed a statistically significant difference between dominant and non-dominant hands in the movement time (Z = −2.255, *p* = 0.024), and mean velocity (Z = −2.549, *p* = 0.011), with non-dominant hands showing longer movement times and velocity (Table 3). However, no significant difference was found in the circle roundness between dominant and non-dominant hands (Z = −0.534, *p* = 0.594).

**Table 3:** Hand difference within the CP group

Movement time (sec)	Z	*p*-value	Median (Dominant hand)	Median (Non-dominant hand)
	−2.25	0.02	4.52	4.61
Mean velocity (mm/sec)	−2.54	0.01	20.39	20.81
Circle roundness	−0.53	0.59	0.90	0.85

### Age-related differences

Participants were stratified into younger (10–13 years) and older (14–17 years) age groups, based on NHS age categorisation guidelines [43]. A Mann-Whitney U test revealed that age group did not significantly influence the movement kinematics of either the dominant or non-dominant hand in younger or older children (Table 4).

**Table 4:** Age-related differences within the CP group

	Younger vs. older (Dominant hand)		Younger vs. older (Non-dominant hand)	
	Mean difference	*P* value	Mean difference	*P* value
Movement time (sec)	0.9	0.62	0.8	0.46
Mean velocity (mm/sec)	14.1	0.58	14.9	0.57
Circle roundness	0.3	0.80	0.2	0.32

### Comparison between the CP and typically-developing groups

An exploratory analysis was conducted using data from a separate study [25], where typically-developing children completed a longer version of the iVR circle drawing task, to compare how typically-developing and CP groups interacted with the task. A Man-Whitney test showed there was no significant difference in the movement time between the CP group and the typically-developing group (U = 121.00, Z = − 1.16, *p* = 0.24 – Table 5). However, results showed that there were significant differences between the two groups in the mean velocity (U = 45.00, Z = − 3.32, *p* = < 0.001) and circle roundness (U = 37.00, Z = − 3.612, *p* = < 0.001), with CP group demonstrating higher velocity but less round circles than the typically-developing group (Table 5).

**Table 5:** Comparison between CP and typically-developing groups

**Descriptive statistics**					
**SUS score**	TD (n = 36)			CP (n = 9)	
	74 ± 11.3			74 ± 16.1	
**VR kinematic data**	Dominant hand (mean±SD) TD vs CP			Non-dominant hand (mean±SD) TD vs CP	
Movement time (sec)	5.28 ± 2.86		4.25 ± 0.95	5.34 ± 2.79	4.38 ± 0.87
Mean velocity (mm/sec)	21.97 ± 10.81		25.12 ± 11.07	25.51 ± 13.03	25.34 ± 11.16
Circle roundness	0.86 ± 0.07		0.75 ± 0.03	0.84 ± 0.07	0.77 ± 0.02
**Between-group differences**					
	U	Z	p-value	Mean rank (TD)	Mean rank (CP)
Movement time (sec)	121.00	−1.16	0.24	24.14	18.44
Mean velocity (mm/sec)	45.00	−3.32	< 0.001	19.75	36.00
Circle roundness	37.00	−3.61	< 0.001	26.47	9.11

*SD* Standard deviation, *TD* Typically developing, *CP* Cerebral palsy

## Discussion

The aim of this study was to examine the usability and acceptability of an iVR circle drawing task to capture the upper-limb motor performance of children and young people with CP. Our data show promising results for this task, delivered through a Meta Quest 2 HMD, which is usable for measuring upper-limb performance in this population. This finding is consistent with that of Phelan, Carrion-Plaza [18] who reported that iVR HMDs are usable as a home-based intervention for different paediatric patients. In addition, the findings from the SUS indicated good levels of usability and acceptability, and the average score of 74 obtained in this study was above the standard mean score of 68 for this scale, and is consistent with our previous work showing high levels of usability and acceptability of iVR-based circle drawing in typically-developing children and young people [25].

The three circle drawing metrics (movement time, mean velocity, and roundness ratio) were not related to perceived usability as assessed by the SUS, which could be explained by the small sample observed in the present study. However, children who drew circles rapidly (velocity) and precisely (roundness) tended to exhibit better unilateral manual ability, as assessed by the BBT. It is important to note that this relationship reflects a within-group association among children with CP rather than a comparison between children with CP and typically developing peers. The average score of the BBT in the present study was 36 blocks, which is higher to that of Liang, Chen [33] who reported an average score of 25 blocks. Our finding is presumably due to our inclusion criteria recruiting only children with mild CP and have good hand functionality. Although children with CP demonstrated higher movement velocity at the group level compared with typically developing children, this should not be interpreted as superior motor performance, but rather as differences in movement strategy or speed modulation. The average time taken to draw circles was unrelated to performance on the BBT, or DHI scores. However, children who scored high on the DHI (indicating more hand impairments) tended to draw circles at lower speed which were less round. The average score of the DHI in the present study was 23, which is similar to that of Sanal-Top, Karadag-Saygi [36] who reported an average score of 22.9.

The findings from the current study are well-aligned with those of Alrashidi, Evans [25], highlighting that iVR circle drawing is usable and acceptable for children and young people. Table 5 compares the mean scores of similar outcome measures of the present study, and the study of Alrashidi, Evans [25]. The descriptive statistics analyses of this table highlights that the performance and scoring of children with CP is slightly lower than their typically-developing peers. Despite the variance in sample sizes between the two studies, these differences could be explained by the motivation, engagement and concentration inherent in VR for children with CP relative to their typically-developing peers [44–46]. In addition, children with CP may perceive it more as a game rather than a diagnostic tool [47] which may enhance further the gap in the performance between the two groups. However, SUS scores between the groups were similar, which could be explained by the fact that, in the present study, 44% (4 out of 9 children) were VR users, whereas in Alrashidi, Evans [25] only 8% (3 out of 36 children) were VR users. Furthermore, it is worth noting that the typically-developing children in Alrashidi, Evans [25] drew 32 circles, whereas participants in the current study drew 16 circles, which could have further narrowed the gap in SUS scoring. However, the findings from the Table 5 (the second part of that table) revealed important differences within the CP group and between the typically-developing and CP groups.

The findings from the Wilcoxon Signed-Rank Test show that there are significant differences between the dominant and non-dominant hands within the CP group in movement time and mean velocity, with dominant hand exhibiting shorter movement times and higher velocities. Comparison of this finding with earlier studies confirms that the dominant hand in CP tends to efficiently perform more controlled movements compared with non-dominant hand [5, 48, 49], which could be attributed to daily use and preferences [50]. Surprisingly, there were no differences in the circle roundness between the two hands, which is an interesting observation showing that both hands were capable of controlling drawing equally smoothly when there are no temporal constraints. Future work could examine whether accuracy asymmetries emerge when the movement times and velocities are matched between the hands, or during a bimanual version of the iVR circle drawing where attention must be divided between the hands [51].

By comparing the CP and typically-developing groups, the results showed that there was no difference in the movement time between the two groups, showing that all children were able to complete the iVR task in a similar duration. However, the CP group showed higher velocity but drew less rounded circles compared to the typically-developing group. It is possible that this finding is related to the children with CP who may prioritize speed over precision, which is a common observation in children with neuromuscular impairments [52]. Alternatively, the children may have motor planning deficits, affecting their ability to slow down and adjust the movement similar to the typically-developing group [52, 53]. Children with CP drew less rounded circles compared to the typically-developing group, which could imply some issues with maintaining a precise trajectory to produce smoother circular movements. This observation is applicable as it shows that children with CP can achieve similar movement times to typically-developing children, but their movement quality could be less refined, leading to lower smoothness and precision. This finding might be explained by the observation that the motor impairments related CP, such as spasticity or motor control deficits, which impact the ability of children to perform controlled circular movements [54].

Although little research has been conducted examining circle drawing in young people, prior studies using this task with children have shown its value in assessing aspects of upper-limb motor function, such as movement smoothness, speed, accuracy, and coordination [20, 22, 23]. However, these studies have employed digitizing tablets to capture the circle drawing and tracing performance of typically-developing children, which leads to questions the applicability and accuracy of methods used in previous studies. For example, the iVR environment offers immersive visual feedback and freehand spatial interaction, which may engage different aspects of motor control [55]. Another factor that potentially influences drawing performance on tablets is that the drawing movement is supported by gravity. In contrast, in the present study, the movement was gravity-independent, which could require participants to exhibit more precision and effort to draw circles. The iVR-based circle drawing used in this study offers an innovative upper-limb motor task with minimal compensatory movements compared with previous methods. A boundary was placed around the VR circle to monitor and prevent participants from making movements that were not task-directed (i.e., drawing upwards away from the circle). This boundary was 7 cm in each direction. If a participant’s movement crossed this boundary, a prompt message requested the participant to redo the trial more accurately. From the perspective of clinical practice, this is critical to ensure that children practise the targeted function precisely, with minimal compensatory recruitment of proximal musculature of the upper-limb [56]. To support this, the task can be adjusted by modifying the size of the drawing space, movement amplitude, or required precision to match each child’s motor abilities. This individualized calibration helps reduce the likelihood of compensatory strategies by ensuring that the task remains challenging but achievable within the child’s functional range. However, future studies using the VR circle drawing task may explore assessment configurations without a visual circle to trace and incorporate deviation-based metrics (e.g., spatial error from the target circle, time spent outside the trajectory, or movement smoothness) to enhance sensitivity to subtle upper-limb motor impairments.

Although the study has a small sample size, there are several important implications for clinical practice and future research. The VR circle drawing task demonstrated its ability to distinguish between the two hands, reflecting the natural asymmetry in motor performance [57, 58]. By capturing hand-specific metrics, the task shows subtle differences in control between the two limbs, further supporting its validity in assessing upper limb motor function. The present study has demonstrated the potential for a range of tasks delivered through VR HMDs in the context of examining upper-limb motor performance of children with CP in an engaging and playful environment. The findings from this study can be used as a first step to further develop and research the nature of VR assessment’s role in paediatric rehabilitation clinical practice. In addition, the findings can be used for direct replication in a larger sample size. However, a few limitations need to be noted regarding the present study. This study is limited by the small sample size, therefore the findings might not be generalisable to the broader clinical population, with a clear need for future clinical research with large sample sizes to understand how VR HMDs can aid in assessing the upper-limb of paediatric patients. The VR-based circle drawing task used in this study was constrained by the fact that it is restricted to children with mild CP and mild cognitive impairments, which would not fully encompass the diverse spectrum of people who live with CP. Additionally, while usability from this study is a positive step, demonstrating the usability of iVR circle drawing task as a clinical tool requires broader evidence and further research, including aspects beyond usability, such as safety, effectiveness, reliability, accessibility, clinical integration, and user experience. In addition, postural demands were not systematically controlled or analysed in the present study, as participants were permitted to perform the task in either sitting or standing positions to ensure comfort and safety. Given the established interaction between postural control and upper-limb motor performance in children with CP [59], future studies should examine the influence of posture on VR circle drawing outcomes under standardised sitting and standing conditions. Furthermore, although children with known visual disorders were excluded based on parent or caregiver report, formal screening for visual impairments, including cerebral visual impairment, was not undertaken. Given the high prevalence of cerebral visual impairment in children with CP and the visually guided nature of immersive VR tasks, future studies should incorporate standardised vision and visual-perceptual screening to better understand the influence of visual function on task performance.

## Conclusions

This study investigated the usability of an iVR circle drawing task in assessing the upper-limb motor function of children with CP. The study indicated that this task is usable for capturing the upper-limb motor performance of children and young people with CP. The iVR circle drawing task captured differences in the movement kinematics of both hands of children with CP, and some of its outcomes correlated well with objective measures of upper-limb performance. Participants reported no adverse effects from the use of the headset, and the protocol demonstrated high levels of usability based on the SUS score (74.3 ± 16.2), indicating good usability according to established interpretive criteria. Future research with larger sample sizes and broader populations is warranted to evaluate the circle drawing task’s feasibility, validity, and reliability.

## Supplementary Information

Supplementary Material 1

Supplementary Material 2

## Data Availability

The datasets used and/or analysed during the current study are available from the corresponding author on reasonable request.

## Abbreviations

CP: Cerebral Palsy
VR: Virtual Reality
iVR: Immersive Virtual Reality
BBT: Box And Block Test
NHS: National Health Services
MACS: Manual Ability Classification System
HMDs: Head-Mounted Displays
GMFCS: Gross Motor Function Classification System
SUS: System Usability Scale
DHI: Duruoz Hand Index
TD: Typically Developing
SD: Standard Deviation
