# Supplementary material for: Usability of a virtual reality circle drawing task to assess upper-limb motor performance in children and young people with cerebral palsy: pilot study
Source: BMC Pediatr. 2026 Feb 24;26:244. doi: 10.1186/s12887-026-06626-8 (PMC13037249; doi:10.1186/s12887-026-06626-8)
Supplement: Supplementary file 1 — Supplementary Material 1 [file 12887_2026_6626_MOESM1_ESM.docx]

**Additional file 1: Frequency table of SUS responses**

| Statements | Strongly disagree | Disagree | Neutral | Agree | Strongly agree |
| --- | --- | --- | --- | --- | --- |
| I think that I would like to use the VR headset frequently. | 0 | 0 | 1 | 4 | 4 |
| I found the VR headset unnecessarily complex. | 3 | 4 | 0 | 2 | 0 |
| I thought VR headset was easy to use. | 0 | 1 | 0 | 2 | 6 |
| I think that I would need the support of a technical person to be able to use the VR headset. | 0 | 6 | 3 | 0 | 0 |
| I found the various functions in the VR headset and environment were well integrated. | 0 | 0 | 9 | 0 | 0 |
| I thought there was too much inconsistency in the VR headset. | 5 | 4 | 0 | 0 | 0 |
| I would imagine that most of my friends would learn to use VR very quickly. | 0 | 0 | 0 | 3 | 6 |
| I found the VR headset very cumbersome (awkward) to use. | 1 | 3 | 4 | 0 | 1 |
| I felt very confident using the VR headset. | 0 | 1 | 0 | 5 | 3 |
| I needed to learn a lot of things before I could get going with the VR headset. | 0 | 4 | 2 | 3 | 0 |
